# Training executive functions using an adaptive procedure over 21 days (10 training sessions) and an active control group

**DOI:** 10.1177/17470218211002509

**Published:** 2021-03-30

**Authors:** Martina De Lillo, Victoria EA Brunsdon, Elisabeth EF Bradford, Frank Gasking, Heather J Ferguson

**Affiliations:** School of Psychology, Keynes College, University of Kent, Kent, UK

**Keywords:** Executive functions, working memory, inhibitory control, cognitive flexibility, cognitive training, transfer effects

## Abstract

The degree to which executive function (EF) abilities (including working memory [WM], inhibitory control [IC], and cognitive flexibility [CF]) can be enhanced through training is an important question; however, research in this area is inconsistent. Previous cognitive training studies largely agree that training leads to improvements in the trained task, but the generalisability of this improvement to other related tasks remains controversial. In this article, we present a pre-registered experiment that used an adaptive training procedure to examine whether EFs can be enhanced through cognitive training, and directly compared the efficacy and generalisability across sub-components of EF using training programmes that target WM, IC, or CF versus an active control group. Participants (*n* = 160) first completed a battery of tasks that assessed EFs, then were randomly assigned to one of the four training groups, and completed an adaptive procedure over 21 days (10 training sessions) that targeted a specific sub-component of EF (or was comparatively engaging and challenging, but did not train a specific EF). At post-test, participants returned to the lab to repeat the battery of EF tasks. Results revealed robust direct training effects (i.e., on trained task), but limited evidence to support near (i.e., same EF, different task) and far (i.e., different EF and task) transfer effects. Where indirect training benefits emerged, the effects were more readily attributable to the overlapping training/assessment task routines, rather than more general enhancements to the underlying cognitive processes or neural circuits.

## Introduction

Executive function (EF) is a commonly used “umbrella term” to describe the set of processes that are responsible for higher level action control (e.g., planning, inhibition, coordination and control of behaviours), and are necessary to maintain specific goals and resist distraction from alternatives. As such, EFs form the basis of our cognitive functioning (Diamond, 2013; Miyake et al., 2000). In this article, we focus on three sub-components of executive functioning (inhibitory control [IC], working memory [WM], and cognitive flexibility [CF]) that contribute differentially to performance on complex executive tasks (Miyake et al., 2000), and evaluate whether these EF abilities can be enhanced through a 21-day training procedure. IC refers to the ability to inhibit a dominant response to focus on a more appropriate response; WM is the ability to retain information for a brief period of time to perform mental operations; and CF is the ability to shift between different tasks or mental sets, and thus allows us to adapt behaviours to changes in the environment.

EFs have a protracted period of development, which begins in early childhood (~2 years old) and continues into young adulthood, with each sub-component of EF developing at its own rate (Diamond, 2002). For example, WM and planning have been shown to develop throughout childhood and into adolescence or early adulthood (e.g., Bishop et al., 2001; Gathercole et al., 2004), whereas CF and IC are thought to reach adult-like levels by age 12 years (e.g., Crone et al., 2006; van den Wildenberg & van der Molen, 2004). Neuroimaging findings support these distinct components of EF, and show that different goal-directed behaviours are subserved by distinct areas of the brain (e.g., Crone et al., 2006; Narayanan et al., 2005). Various clinical and neuro-degenerative conditions lead to impairments in EFs (e.g., Orellana & Slachevsky, 2013; Schmitt et al., 2018; Stopford et al., 2012), and even healthy ageing is associated with cognitive decline, which leads to general deficits in processing speed as well as specific impairments in EF, including WM (Braver & West, 2008), IC (Maylor et al., 2011), and CF (Greenwood, 2007). Indeed, even when EFs are at their peak, there exists a great deal of individual variation in performance (Carroll & Maxwell, 1979). Understanding the conditions under which cognitive capacities function optimally, and how they relate to each other, is an important question.

Over the last decade or so, researchers have attempted to explore the degree to which EFs can be trained. The potential benefits of cognitive training have been linked to the concept of neuroplasticity. That is, through practice, the brain reshapes its organisation, creating new neural connections, or strengthening existing ones, which leads to reinforcement of the trained cognitive capacity and/or related abilities that were not trained directly (e.g., Ballesteros et al., 2018; Han et al., 2018; Jolles et al., 2013; Kraft, 2012). Cognitive training is therefore based on the underlying principle that training on a specific task leads to improvements across the trained cognitive domain, and that this improvement might also extend to other related cognitive domains that were not trained. *Three* different classes of training effects have been identified (Carroll, 1993). *Direct* effects describe an improvement in performance on the trained measure, *near-*transfer effects describe enhanced performance on a different measure of the trained construct (e.g., training and assessing WM using different tasks), and *far*-transfer effects describe enhanced performance on a different construct (e.g., training WM and assessing IC). To obtain a transfer, it seems necessary that the trained task and the transfer task involve the same processes. Therefore, transfers are expected when there is overlap between the underlying processes involved in the different tasks (Buschkuehl et al., 2012; Dahlin et al., 2008; Lustig et al., 2009).

To date, findings on whether EF abilities can be improved through training have been mixed (see Diamond & Ling, 2016, and Simons et al., 2016, for a review). On one hand, positive effects of direct training seem to be relatively uncontroversial, with research consistently showing that performance on a specific EF task improves with repeated practice (e.g., McKendrick et al., 2014). On the other hand, the degree to which training effects transfer to untrained tasks or domains remains inconclusive. Some studies have reported near-transfer effects within an EF domain (e.g., Heinzel et al., 2016; Karbach & Kray, 2009; Sandberg et al., 2014), e.g., Heinzel et al. (2016) showed improvements in WM when training and assessment was conducted using different paradigms, and Byrne et al. (2020) showed improvements in WM when training and assessment used different materials but the same task. Others have shown far-transfer effects between EF domains (e.g., Borella et al., 2010; Dowsett & Livesey, 2000; Karbach & Kray, 2009; Salminen et al., 2012), e.g., Karbach and Kray (2009) reported improvements in WM after training in CF. However, many studies have reported no transfer effects at all (e.g., Blacker et al., 2017; Holmes et al., 2019; Melby-Lervåg et al., 2016; Owen et al., 2010; Redick et al., 2013), e.g., WM training did not enhance fluid intelligence in Redick et al. (2013). In fact, while one recent meta-analysis found small but significant improvements in fluid intelligence following WM training (Au et al., 2015), three others have concluded that near-transfer effects following training are weak and short lived, and do not generalise across the sub-components of EF (Melby-Lervåg & Hulme, 2013; Simons et al., 2016; Soveri et al., 2017). Even more curiously, positive effects of cognitive training have been attributed by some researchers to placebo effects, where overtly advertising a study as examining the benefits of cognitive training biases participants’ expectations and subsequent performance relative to participants who were recruited using non-suggestive advertising (Foroughi et al., 2016).

It is notable that the majority of research in this area so far has focused on outcomes following training in a single domain of EF, usually focusing on WM due to its well-known role in cognition, and because it is often impaired in developmental populations (Kray et al., 2012; Pelegrina et al., 2015) and shows a rapid decline in older age (e.g., Heinzel et al., 2014; Jaeggi et al., 2010; Owen et al., 2010; Redick et al., 2013; Richmond et al., 2011). Much less research has tested outcomes following training in other domains of EF, including IC and CF, and studies have very rarely compared training effects directly between different domains of EF. Studies that have tested training effects in these other domains of EF have shown specific performance improvements in tasks related to training, but much less evidence for far-transfer improvements (e.g., Berkman et al., 2014; Enge et al., 2014; Karbach & Kray, 2009; Thorell et al., 2009). It is theoretically interesting to directly compare training effects following programmes that focus on WM, IC, or CF because all these EFs sustain our cognitive functioning, and while previous research has shown that EF processes moderately correlate with each other (i.e., unity), they also represent distinct (i.e., diversity) processes (Friedman et al., 2006; Miyake et al., 2000). In addition, by conducting a single study that compares outcomes across different EF training tasks, we had better control over key methodological factors that limit conclusions that can be drawn by comparing results from different training studies.

Variability in methodology used across studies is a controversial aspect of research on cognitive training that has limited definitive conclusions on the efficacy of EF training to date. These concerns have prompted leading researchers to make clear recommendations on the optimal approaches for EF training programmes (Diamond & Ling, 2016; Simons et al., 2016). First, it is important to assess baseline cognitive abilities before the training intervention (as well as after) so that the causal effects of training can be accurately quantified, and baseline differences between groups can be controlled. Second, training programmes should include an active control group for comparison with the experimental group(s). Early studies in this area tended to use a passive control group, whose only contact with the experimenters was during the pre- and post-assessment sessions (e.g., Chein & Morrison, 2010; S. C. Li et al., 2008; Salminen et al., 2012), or engaged control participants in an activity that did not match the cognitive demands of the experimental group (e.g., watching a film, playing videogames; Borella et al., 2010; Buschkuehl et al., 2008). Including an active control group allows researchers to rule out social or motivational factors that might elicit differences in performance between groups. Specifically, it controls for potential improvements that might be due to expectations of better performance on the second assessment or higher motivation due to social contact during the training period. Third, it is recommended to use adaptive training programmes in which task difficulty increases as performance improves because this challenges each participant to their own limits and rules out the possibility that a lack of improvement could reflect an insufficiently demanding training procedure. Numerous studies have found superior training effects when the training task difficulty was adaptive (e.g., Brehmer et al., 2012, 2016; Enge et al., 2014; Klingberg et al., 2002; Lövdén et al., 2010; but also see Von Bastian & Eschen, 2016). Indeed, some researchers have used a non-adaptive training protocol as the control group, which presents no cognitive challenge for participants and therefore might be confounded by low motivation in this group. Finally, studies should include large samples of participants who are randomly assigned to control/experimental groups, and have comparable expectations for improvement across groups.

In this article, we present a pre-registered experiment that used a 21-day adaptive training procedure to examine whether EFs can be enhanced through cognitive training, and for the first time directly compared the efficacy and generalisability across sub-components of EF of training programmes that target WM, IC, or CF versus an active control group. Specifically, we compared performance on a battery of EF assessments before and after training to test for direct training effects (i.e., improvement on the trained task), near-transfer effects (i.e., improvement on a different task that measures the same construct), and far-transfer effects (i.e., improvement on a different task that measures a different construct). We specifically chose assessment tasks that differed in both paradigm and stimuli from the training task in each sub-component of EF, to ensure that any indirect training effects could not be attributed to shared strategies or response requirements between tasks. Training consisted of 10 sessions over an average of 21 days, which participants completed at home through an online platform, with each training session lasting ~15 min (based on Enge et al., 2014; Zinke et al., 2014). Importantly, we used an active control group, in which participants completed a comparatively engaging and challenging task (an adaptive version of the lexical decision [LD] task) for the same duration as the EF training groups, and were blind to the different groups being tested. We tested a large sample (*n* = 160 participants; 40 in each training group), and randomly assigned each participant to one of the four training groups.

Based on previous research summarised in Simons et al. (2016), we predicted that direct training effects would be apparent in all four training groups, i.e., performance on the trained task would improve from pre- to post-training. We also expected to observe small effects of near transfer in the three EF training groups, i.e., performance in the tasks that measured the same construct as the trained EF would improve from pre- to post-training. Finally, we tested whether training would lead to far-transfer effects in the three EF training groups, i.e., performance in the tasks that measured a different cognitive construct to the trained EF would improve from pre- to post-training. We did not expect to find any far-transfer training benefits in the control group, i.e., no improvement on any of the EF tasks from pre- to post-training.

## Methods

All methodological procedures were pre-registered on the Open Science Framework (OSF) web pages (https://osf.io/5huc8). We note that pre-registration was submitted part-way through data collection and prior to any analysis.

### Participants

A total of 299 participants, aged between 18 and 35 years old, were recruited from the student population at the University of Kent, UK. Of this total sample, 37 participants were excluded because they did not complete the online training sessions appropriately (i.e., under-training: less than nine sessions completed, or over-training: 12 or more sessions completed), 78 participants did not return to complete the post-training assessments, 16 participants were excluded as they were not native English speakers, and a further eight participants were excluded due to technical problems saving data. All participants were native English speakers, had normal or corrected-to-normal vision, had no known neurological disorders, and had no mental health or autism spectrum disorder diagnoses. Participants were randomly assigned to one of the four training groups, with the final sample of *n* = 160 equally split between the four training groups (see Table 1 for demographic details per group), consistent with our pre-registered target sample size. The target sample of *n* = 160, 40 per group, was chosen a priori based on similar research (e.g., Enge et al., 2014; Zinke et al., 2014), and a post hoc power calculation showed that this sample yielded an estimated power of 87% with the significance level of α = .05 on 80% of occasions (as suggested by J. Cohen, 1988). Participants’ consent was obtained according to the Declaration of Helsinki, and the Ethical Committee of the School of Psychology, University of Kent, approved the study.

**Table 1.** Participant demographics for each training group.

Training group	*n*	*M* age (*SD*)	M:F
Inhibitory control	40	19.6 (3.9)	7:33
Working memory	40	20.2 (3.4)	7:33
Cognitive flexibility	40	19.2 (2.3)	3:37
Control group	40	19.3 (2.0)	5:35

### Materials

#### Pre- and post-assessment tasks

All participants completed three assessment tasks in the lab during the pre- and post-training sessions, approximately 21 days apart.

##### Operation span

This task was used to measure WM (Unsworth et al., 2005). Replicating the task used by Unsworth et al. (2005), participants were asked to remember a sequence of letters that appeared one at a time on the computer screen (F, H, J, K, L, N, P, Q, R, S, T, and Y). Between each letter, there was a distractor task (an arithmetical problem to solve). Participants were asked to recall the letters in the correct order at the end of each trial, clicking a box next to the appropriate letter(s) presented in a 4 × 3 matrix. A number appeared in the clicked box to indicate the order, and after completing the sequence of letters, participants received feedback on the correct number of the letters recalled. In cases where participants were not able to recall one or more letters, they were instructed to click on a blank box. Before the main task, participants familiarised themselves with the task through three practice blocks. The first block presented single letters in the middle of the screen for 800 ms, and participants had to memorise sequences of two or three letters. The second practice block required participants to solve some maths equations (e.g., [2 × 1] + 1 = 3) by indicating whether the answer was correct or incorrect as quickly and accurately as possible. In the last practice block, participants completed both the letter recall and maths tasks together. First, the maths equation was presented, followed by a letter appeared in the middle of the screen for 800 ms; this sequence was repeated twice to create two-letter span trials, then the letter recall screen with the 4 × 3 letter matrix was presented. Participants completed three full practice trials, and were given feedback on how many letters they recalled correctly and how many errors they made on the maths problems. After completing the practice blocks, they started the experimental block which consisted of three trials for each of 2 to 7 letter spans (in a randomised order for each participant). This created a total of 18 trials with 81 maths problems and 81 letters. Participants were encouraged to keep their maths accuracy at or above 85% at all times. During recall, a percentage in red was presented in the upper right-hand corner of the screen, indicating the percentage accuracy for the maths problems. The dependent variable for this task was the *Partial Ospan Score*, calculated as the total number of letters correctly recalled, regardless of order.

##### Stroop task

This task was used as a measure of IC (Stroop, 1935). Participants were shown a series of words on the screen in one of the four colours: RED, BLUE, GREEN, and YELLOW. Colour words (e.g., BLUE) were presented in either a consistent or inconsistent colour (i.e., the word BLUE shown in blue/red ink, respectively). Neutral words were also presented (e.g., CAT printed in green ink) to provide a baseline of colour naming without lexical interference. Participants were instructed to respond as quickly and accurately as possible to the ink colour of the words, ignoring the meaning, using a keyboard. Four coloured stickers indicated the four different colour responses: RED, BLUE, GREEN, and YELLOW. Once the word appeared on the screen, participants gave their response and the next trial started immediately. After completing a practice block of 20 trials (10 neutral and 10 congruent), participants completed the experimental block which consisted of 50 congruent trials, 50 incongruent trials, and 50 neutral trials. A blank screen appeared for 1000 ms at the start of the experimental trials. After the participant made a response, the next trial appeared immediately. Words were presented in a pseudo-randomised order, in which the same colour word, the same printed ink colour, or the same colour word/ink colour combination could not appear on two consecutive trials to avoid priming effects. We measured accuracy and reaction times (RT) for neutral, congruent, and incongruent trials. For analysis, we calculated a *Stroop Effect* score for correct responses only; response times were first transformed to *z* scores, and the Stroop effect was calculated by subtracting the mean RT for congruent trials from the mean RT for incongruent trials.

##### Wisconsin Card Sorting Task

This task was used to measure CF (Grant & Berg, 1948; Miyake et al., 2000). Participants were asked to sort cards according to one of the three classification rules: colour (red, blue, yellow, or green), shape (crosses, circles, triangles, or stars), or number of symbols (one, two, three, or four). A series of four cards appeared on the top of the screen which differed in colour, shape, or number of symbols, and one card appeared at the centre bottom. Participants had to figure out which of the three possible sorting rules to adopt according to the feedback that they received after choosing a card. Participants were told that the sorting rule would change throughout the task. There was no practice block, and the experimental block consisted of 128 cards. The task started immediately by presenting four cards on the top of the screen and one on the bottom. After clicking on a card, feedback was displayed on the screen stating whether the card had been sorted correctly or incorrectly. If incorrect feedback was received, participants had to switch to a different rule until they received correct feedback. After 10 consecutive correct trials, the rule changed. The next card appeared immediately after clicking on one of the four cards on the top of the screen. The dependent variable was the total number of perseverative errors, defined as the number of times in which participants persisted with an incorrect sorting rule.

#### Training tasks

Participants were randomly assigned to one of the four groups, three that trained a specific component of EF (WM, IC, or CF), and an active control group (LD task). The training tasks were designed to be adaptive, in that task difficulty increased/decreased based on the participant’s performance. Specifically, accuracy was monitored for each block so that if a participant’s accuracy on that block equalled or exceeded 90%, the task moved up to the next level of difficulty, and if accuracy equalled or fell below 75%, the task returned to the previous level of difficulty. When accuracy on a block fell between 76% and 89%, participants repeated the same level. If participants achieved the maximum level of difficulty or the minimum level of difficulty with accuracy below 75%, they repeated the same level on the next block. Details of the levels of difficulty used for each of the training tasks are provided below. Participants received feedback on their accuracy at the end of each block. Practice blocks were excluded for training sessions completed at home. Training tasks were completed online. Each training session lasted approximately 15 min.

##### *N*-back task

WM training adopted a visual version of the *n*-back task (J. D. Cohen et al., 1993). A series of letters appeared one by one in the centre of the screen (500 ms), and participants’ task was to press a button on the keyboard if the letter presented was the same as the one presented *n* trials before. No response was required if the letter did not match. There were six different *n*-back levels: 1-, 2-, 3-, 4-, 5-, and 6-back (e.g., in the 2-back condition, participants should respond if the current letter is the same as the letter presented two trials before). Participants first completed three practice blocks with 1-, 2-, and 3-back, and then completed a further 15 blocks in the lab task or 21 blocks in the online task. Each block included 20 trials, with a fixed ratio of target/non-target trials of 6/14. Task difficulty increased over 15 levels by manipulating the *n*-back levels (between 2- and 6-back) and interstimulus interval (ISI; 1,800; 1,600; and 1,400 ms), as shown in Table 2. The dependent variables for this task were average level and accuracy, calculated as the proportion of Hits (i.e., correctly identifying a target as a target) minus False Alarms (i.e., incorrectly classifying a non-target as a target).

**Table 2.** Difficulty levels used in the *n*-back (WM) training task.

Level	*n*-back	ISI (ms)	Level	*n*-back	ISI (ms)	Level	*n*-back	ISI (ms)
1	2-back	1,800	6	3-back	1,400	11	5-back	1,600
2	2-back	1,600	7	4-back	1,800	12	5-back	1,400
3	2-back	1,400	8	4-back	1,600	13	6-back	1,800
4	3-back	1,800	9	4-back	1,400	14	6-back	1,600
5	3-back	1,600	10	5-back	1,800	15	6-back	1,400

ISI: interstimulus interval; WM: working memory.

##### Stop signal-flanker task

IC training adapted the stop signal task (SST; Logan, 1994) used in Berkman et al. (2014). Participants were presented with a black arrow in the centre of the computer screen, pointing left or right. Participants’ task was to press the left or right arrow key on the keyboard to indicate the direction of this central arrow. However, on 25% of trials, the arrow turned red, after a variable stop signal delay (SSD). Participants were instructed to withhold their response on these trials. The task involved three short practice blocks, followed by nine blocks of experimental trials. Each block included 44 trials, with an equal ratio of left/right-facing arrows. Task difficulty increased over eight levels by manipulating the presence and features of flanker stimuli and the length of the SSD (50–250 ms and 300–500 ms), as shown in Table 3. When flanker stimuli were present (Levels 3–8), two additional arrows were placed either side of the central target arrow, and could either face the same direction as the central arrow (levels 3 and 4) or faced in a different direction to the central arrow (Levels 5–8). On Levels 3–6, these flanker stimuli could either be black or red, and did not change colour during the trial. Participants were instructed to ignore these distractor arrows and only respond to the direction and colour of the central arrow. An additional rule was added for the final two levels, as the arrows could appear in black, red, or blue ink, and participants were instructed to withhold a response only for red colour changes to the central arrow (ignoring flanker arrows and responding to blue colour changes to the central arrow). Thus, this task assessed two IC processes: the ability to withhold a response and the ability to ignore competing stimuli (that may conflict with the target). The dependent variables for this task were average level and accuracy, calculated as the proportion of Hits (i.e., correctly identifying a black/blue arrow as a target) minus False Alarms (i.e., incorrectly classifying a red arrow as a target).

**Table 3.** Difficulty levels used in the stop signal-flanker (IC) training task.

Level	Trial type	SSD (ms)	Example stimuli
1	Single arrow	50–250	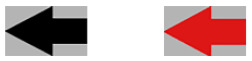
2	Single arrow	300–500	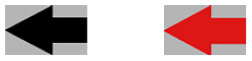
3	Flanker arrows (same direction)	50–250	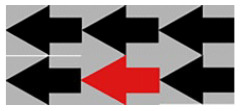
4	Flanker arrows (same direction)	300–500	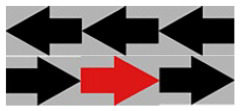
5	Flanker arrows (different direction)	50–250	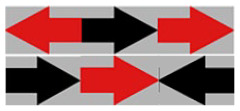
6	Flanker arrows (different direction)	300–500	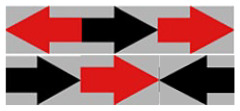
7	Flanker arrows (different direction, additional colour)	50–250	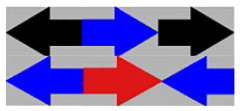
8	Flanker arrows (different direction, additional colour)	300–500	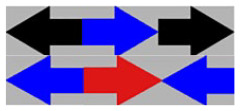

IC: inhibitory control; SSD: stop signal delay.

##### Task switching

CF training adapted the task switching (Rogers & Mansell, 1995) paradigm used in Barenberg et al. (2015). Participants were presented with a 2 × 2 grid on the computer screen, and bivalent stimuli (a circle or triangle, in blue or yellow colour) appeared one by one in each of the four quadrants. Participants’ task was to classify the stimuli by colour or shape, depending on trial type, using the keyboard. The task involved a short practice block, followed by 19 blocks of experimental trials. Each block included 32 trials, with an equal ratio of shape/colour combinations. Task difficulty increased over 12 levels by manipulating trial type (from single-task to mixed-task) and ISI (1,250; 1,000; and 800 ms), as shown in Table 4. In the single-task trial type, participants had to identify whether the stimuli colour was blue or yellow (Levels 1–3), or whether the shape was a circle or triangle (Levels 4–6). In the mixed-task trial type, participants indicated the stimuli’s shape when it appeared in the upper two quadrants and the stimuli’s colour when it appeared in the lower two quadrants (thus had to switch categorisation rule). Stimuli either appeared in a predictable clockwise manner (Levels 1–9) or appeared in an unpredictable location in the grid (Levels 10–12). The dependent variables for this task were average level and accuracy, calculated as the proportion of Hits (i.e., correctly identifying a target feature) minus False Alarms (i.e., incorrectly classifying a target feature).

**Table 4.** Description of the levels in the task switching training protocol.

Level	Trial type	Stimuli presentation	ISI (ms)
1	Single-task (colour)	Clockwise	1,250
2	Single-task (colour)	Clockwise	1,000
3	Single-task (colour)	Clockwise	800
4	Single-task (shape)	Clockwise	1,250
5	Single-task (shape)	Clockwise	1,000
6	Single-task (shape)	Clockwise	800
7	Mixed-task	Clockwise	1,250
8	Mixed-task	Clockwise	1,000
9	Mixed-task	Clockwise	800
10	Mixed-task	Unpredictable	1,250
11	Mixed-task	Unpredictable	1,000
12	Mixed-task	Unpredictable	800

ISI: interstimulus interval.

##### LD task

For the active control condition, we adopted a task that would be sufficiently cognitively taxing for participants, but would not train any specific EF ability: the LD task (Meyer & Schvaneveldt, 1971). In this task, participants used the keyboard to classify strings of letters as a word or non-word. A total of 3,984 words were obtained using the MRC Psycholinguistics Database (Wilson, 1988) and were categorised according to their word frequency: high frequency (HF), middle high frequency (MHF), middle low frequency (MLF), and low frequency (LF). Using the Wuggy pseudoword generator, 7,968 non-words were generated (Keuleers & Brysbaerd, 2010), retaining either one or two syllables from the matched real word (e.g., compare—cobbane—combore). The task involved a short practice block, followed by nine blocks of experimental trials. Each block included 40 trials, with an equal ratio of words and non-words, and each word was presented for 3,000 ms. Task difficulty increased over eight levels by manipulating word frequency (from high to low frequency) and the number of retained syllables for non-words (from one to two), as shown in Table 5. The dependent variables for this task were average level and accuracy, calculated as the proportion of Hits (i.e., correctly identifying a word as a word) minus False Alarms (i.e., incorrectly classifying a non-word as a word).

**Table 5.** Description of difficulty levels in the lexical decision training task.

Level	Word frequency	Retained syllables	Example word/non-word
1	HF	1	Activity/Oupevici
2	HF	2	Activity/Aupetity
3	MHF	1	Compare/Cobbane
4	MHF	2	Compare/Combore
5	MLF	1	Expedient/Asquadent
6	MLF	2	Expedient/Ertopient
7	LF	1	Villainous/Nuttoilous
8	LF	2	Villainous/Nellailous

HF: high frequency; MHF: middle high frequency; MLF: middle low frequency; LF: low frequency.

#### Motivation assessment

At the end of each online training session, participants completed a short questionnaire to assess their motivation to complete the task. This questionnaire was based on the Intrinsic Motivation Inventory (Deci & Ryan, 2010) and consisted of six statements (e.g., “I enjoyed doing this activity very much,” “I found this activity hard to complete”), which participants rated on a Likert-type scale from 0 (*not at all true*) to 7 (*very true*). Scoring was reverse coded where necessary to ensure that higher scores indicated greater motivation for each statement. An average motivation score, across all six statements and 10 online training sessions, was calculated for each participant.

### Procedure

Participants first completed the 45-min pre-training session in the lab, which included the three assessment tasks in a randomised order (i.e., OSpan, Stroop, and Wisconsin Card Sorting Task [WCST]), followed by their assigned training task (either *n*-back, stop-signal flanker, task switching, or LD). At the end of the pre-training session, they received instructions on the procedures to complete the online training at home. Participants were invited to complete 10 online training sessions at home, each lasting ~15 min, over the next 21 days. From the final sample, 43 participants completed only nine training sessions at home (IC = 7, NB = 17, CF = 5, LD = 14) and five participants completed 11 training sessions at home (CF = 4, LD = 1). Training tasks were controlled through INQUISIT software (www.millisecond.com), and participants were sent personalised emails with a link to the appropriate task every 2 or 3 days. Participants started each training session from the first level of difficulty. Following the 21-day training period,^1^ participants returned to the lab to complete the post-training session, in which they repeated the same three assessment tasks from the pre-training session, as well as their assigned training task. Data were collected over a period of 24 months, between 2017 and 2019, and participants received university credits for participating.

## Results

All analysis procedures were pre-registered, and the full datasets and analysis scripts are available on the OSF web pages (https://osf.io/whxvt/). Note that pre-registration was submitted part-way through data collection, and an amended analysis plan was submitted after full data collection but prior to any analysis (i.e., the revised plan ensured greater degree of specificity on how *z* scores should be calculated and comparability between assessment dependent variables [DVs]). All statistical analyses were conducted in R version 3.6.1. The dependent variables were *z*-scored for ease of comparison between tasks.

### Direct training effects

**Figure 1.**
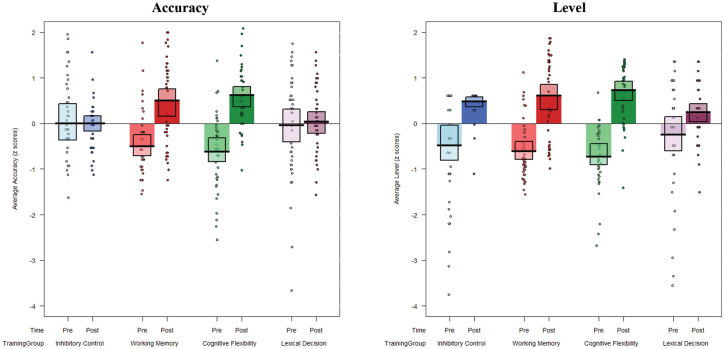
Average *z*-scored accuracy (left panel) and level (right panel) pre- and post-training, plotted for each training group. Accuracy for each task is calculated as proportion of Hits *minus* False Alarms.

To test our first hypothesis of performance improvements in all trained tasks when comparing pre- and post-training sessions, we conducted two mixed analyses of variance (ANOVAs) (one for accuracy and one for level), crossing the within-subjects variable Time (pre- vs. post-training) with the between-subjects variable Training group (WM vs. IC vs. CF vs. LD). Each dependent variable was *z*-scored over pre- and post-training, separately for each Training group. Data for accuracy and level are plotted, separately for each training group and pre-/post-training session, in Figure 1. Data from each of the 12 training sessions in each training group are provided in the online Supplemental material for illustration.

#### Accuracy

Results revealed a significant main effect of Time, *F*(1, 312) = 31.08, *p* < .001, $\eta_{p}^{2}$ = .09, reflecting improved overall performance from pre- to post-training. Moreover, the interaction between Time and Training group was significant, *F*(3, 312) = 9.45, *p* < .001, $\eta_{p}^{2}$ = .08, suggesting that training effects differed between the four groups from pre- to post-training. To examine this interaction further, and following the pre-registered analysis, follow-up *t* tests were conducted on pre-training versus post-training outcomes separately for each group. Post hoc tests showed that accuracy improved significantly from pre- to post-training in the WM, *t*(39) = 7.16, *p* < .001, *d* = 1.13, and CF, *t*(39) = 8.25, *p* < .001, *d* = 1.56, training groups, but did not improve significantly from pre- to post-training in the IC, *t*(39) = 0.03, *p* = .97, *d* < .01, or LD, *t*(39) = 0.42, *p* = .67, *d* = .07, control groups.

#### Level

Results revealed a significant main effect of Time, *F*(1, 312) = 120.72, *p* < .001, $\eta_{p}^{2}$ = .28, reflecting improved overall performance from pre- to post-training. Moreover, the interaction between Time and Training group was significant, *F*(3, 312) = 4.73, *p* = .003, $\eta_{p}^{2}$ = .04, suggesting that training effects differed between the four groups from pre- to post-training. To examine this interaction further, and following the pre-registered analysis, follow-up *t* tests were conducted on pre- and post-training outcomes separately for each group. Post hoc tests showed that the average of level difficulty improved significantly from pre- to post-training in all four groups—IC: *t*(39) = 4.73, *p* < .001, *d* = 1.11; WM: *t*(39) = 9.74, *p* < .001, *d* = 1.48; CF: *t*(39) = 13.75, *p* < .001, *d* = 2.13; LD: *t*(39) = 2.94, *p* = .005, *d* = .48—but was larger in the three EF training groups compared with the control group.

### Indirect training effects

First, we conducted a series of 1-way ANOVAs with Training group as the between-subjects factor to compare pre-training performance on each assessment task across the four training groups. The main effect of Training group was not significant on any measure (see Table 6), indicating that the four training groups did not differ in baseline performance before the training intervention.

**Table 6.** Statistical effects comparing baseline performance on each assessment task between the four training groups.

Assessment task	*df*	*F*	*p* value
OSpan	3, 156	.44	.73
Stroop	3, 156	.43	.73
WCST	3, 156	.49	.69

WCST: Wisconsin Card Sorting Task.

**Figure 2.**
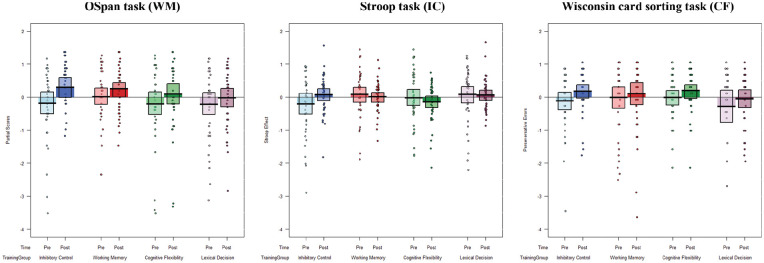
Average *z*-scored partial scores on the OSpan task (left panel), Stroop effect on the Stroop task (middle panel), and perseverative errors on the WCST (right panel) at pre- and post-training, plotted for each training group.

Next, a series of ANOVAs were conducted on each of the assessment tasks to examine indirect training effects (i.e., near and far transfer), crossing the within-subjects variable Time (pre- vs. post-training) with the between-subjects variable Training group (WM vs. IC vs. CF vs. LD). Follow-up *t* tests were conducted to examine pre- and post-training performance in each of the assessment tasks separately for each training group. As per our hypotheses, these analyses examined near- and far-transfer effects of the trained cognitive ability. The perseverative errors for the WCST and the congruency effect for the Stroop task were reverse-scored so that a higher value indicates better performance for all assessment tasks. Data for each assessment task are plotted, separately for each training group and pre/post-training session, in Figure 2.

#### OSpan/WM

Results showed a significant effect of Time, *F*(3, 156) = 21.36, *p* < .001, $\eta_{p}^{2}$ = .12, indicating that participants were more accurate (i.e., had higher partial scores) in the post-training session (*M* = 68.6) compared with the pre-training session (*M* = 65.4). The main effect of Training group was not significant, *F*(3, 156) = 0.62, *p* = .60, $\eta_{p}^{2}$ = .005, thus overall recall accuracy was comparable between the four training groups. The interaction between Time and Training group was also not significant, *F*(3, 156) = 0.91, *p* = .442, $\eta_{p}^{2}$ = .017. These results suggest that performance on the OSpan improved from pre- to post-training regardless of training group.

Following the pre-registered analysis, follow-up *t*-tests were conducted to compare pre- and post-training outcomes separately for each training group. For the WM training group, results showed a non-significant improvement in partial scores from pre- to post-training, *t*(39) = 1.90, *p* = .065, *d* = .30, indicating no significant near transfer from WM training to another measure of WM. However, OSpan partial scores significantly improved from pre- to post-training in both the IC, *t* (39) = 3.83, *p* < .001, *d* = .48, and CF, *t* (39) = 2.17, *p* = .030, *d* = .27, training groups. This suggests that training in IC and CF might have led to far-transfer improvement on a measure of WM. Finally, there was no significant difference in OSpan partial scores from pre- to post-training in the LD training group, *t*(39) = 1.44, *p* = .157, *d* = .20, indicating no improvement in WM in this active control group.

In addition, we ran exploratory analyses that directly compared the difference in partial scores for post- and pre-training (post *minus* pre) session for each of the EF training groups with the control group. Results showed that none of the pre-post partial scores differed between each training group and the control group (all *p*s > .13). Taken together, although OSpan partial scores significantly improved from pre- to post-training following training in IC and CF, this difference was not greater than the practice-based improvement in the control group, thus it is unlikely that far transfer occurred.

#### Stroop/IC

Results revealed no significant effect of Time, *F*(1, 156) = 0.058, *p* = .810, $\eta_{p}^{2}$ < .01, indicating that the Stroop effect did not change from pre- to post-training. There was no significant main effect of Training group, *F*(3, 156) = 0.878, *p* = .454, $\eta_{p}^{2}$ *=* .02, and no significant interaction between Time and Training group, *F*(1, 156) = 1.46, *p* = .227, $\eta_{p}^{2}$ *=* .03. These results suggest that the Stroop effect did not change between pre- and post-training sessions, and did not differ between the four training groups.

Following the pre-registered analysis, follow-up *t*-tests were conducted to compare pre- and post-training outcomes separately for each training group. For the IC training group, results showed a non-significant improvement in Stroop effects from pre- to post-training, *t*(39) = 1.77, *p* = .085, *d* = .35, indicating no significant near transfer from IC training to another measure of IC. There were no significant differences in Stroop effects between pre- and post-training for either the CF, *t*(39) = 0.89, *p* = .38, *d* = .17, or WM, *t*(39) = 0.55, *p* = .585, *d* = .11, training groups, indicating no far transfer from CF or WM training to a measure of IC. Finally, there was no significant difference in the Stroop effect from pre- to post-training in the LD training group, *t*(39) = 0.11, *p* = .915, *d* = .03, thus IC did not improve in this active control group.

In addition, we ran exploratory analyses that directly compared the difference in Stroop effects for post- and pre-training session (post *minus* pre) for each of the EF training groups with the control group. Results showed that none of the pre-post Stroop effect scores differed between each training group and the control group (all *p_s_* > .20).

#### WCST/CF

Results revealed a significant main effect of Time, *F*(1, 156) = 5.45, *p* = .021, $\eta_{p}^{2}$ = .034, indicating that participants made significantly less preservative errors post-training (*M* = 5.04) compared with pre-training (*M* = 6.19). The main effect of Training group was not significant, *F*(3, 156) = 0.79, *p* = .503, $\eta_{p}^{2}$ = .029, indicating that perseveration errors did not differ between the four training groups, and the interaction between Time and Training group was also not significant, *F*(3, 156) = 0.17, *p* = .916, $\eta_{p}^{2}$ = .003. Taken together, these results show that performance on the WCST improved from pre- to post-training, regardless of training group.

Follow-up *t* tests showed a non-significant improvement in perseverative errors between pre- and post-training for the CF training group, *t*(39) = 1.78, *p* = .082, *d* = .28, indicating no significant near transfer from CF training to another measure of CF. There was also no significant difference in perseverative errors between pre- and post-training for either the IC training group, *t*(39) = 1.84, *p* = .073, *d* = .38, or the WM training group, *t*(39) = 0.67, *p* = .504, *d* = .11, indicating no far transfer from IC or WM training to a measure of CF. Perseverative errors also did not differ from pre- to post-training in the LD training group, *t*(39) = 0.92, *p* = .364, *d* = .17, indicating no improvement in this measure of CF in the active control group.

In addition, we ran exploratory analyses that directly compared the difference in perseverative errors for post- and pre-training session (post *minus* pre) for each of the EF training groups with the control group. Results showed that none of the pre-post perseverative error scores differed between each training group and the control group (all *p*s > .71).

#### Motivation assessment

**Figure 3.**
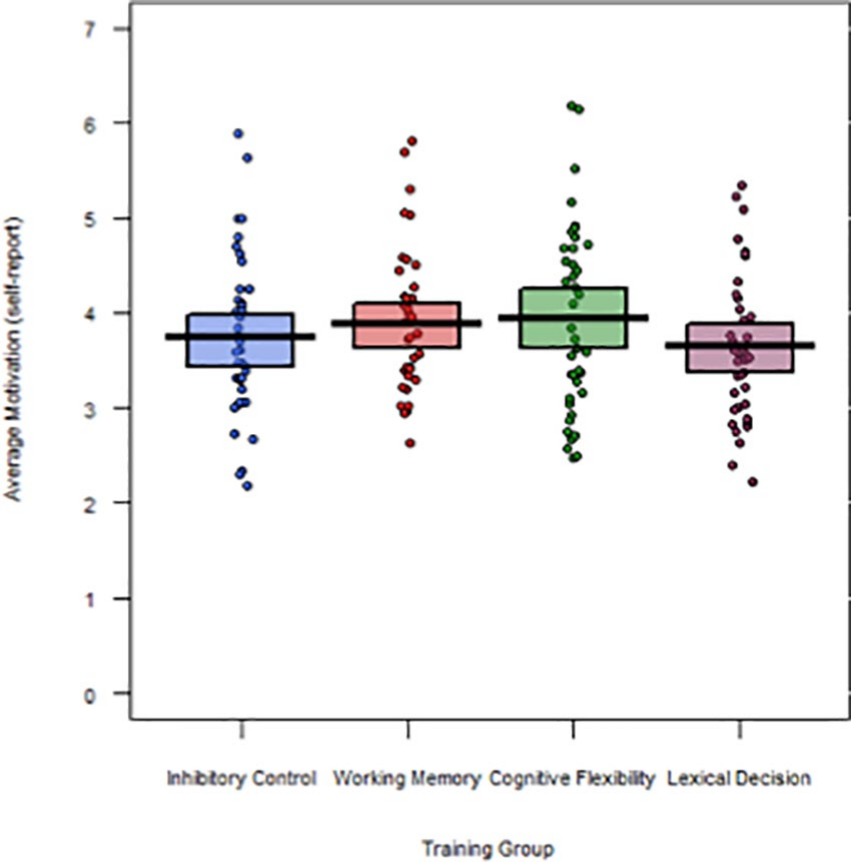
Average self-reported motivation across the 10 online sessions in each training group.

To check for group differences in motivation during the training, we conducted a one-way ANOVA on the questionnaire ratings (averaged over the six statements and 10 online sessions for each participant), with training group as the between-subjects factor. Note that data were missing for one participant (in the LD training group); thus, analyses were conducted on a final sample of 159 participants. As can be seen in Figure 3, results revealed a relatively moderate level of motivation among participants (overall *M* = 3.8), and importantly, there was no difference in motivation between the training groups, *F*(3, 155) = 0.99, *p* = .41, $\eta_{p}^{2}$ *=* .02 (IC: *M* = 3.73, *SD* = .85; WM: *M* = 3.88, *SD* = .77; CF: *M* = 3.93, *SD* = .98; LD: *M* = 3.64, *SD* = .78).

## Discussion

In this pre-registered experiment, we sought to examine whether EFs can be enhanced through cognitive training, and directly compared the efficacy and generalisability of training programmes that targeted WM, IC, or CF versus an active control group. Participants (*n* = 160) first completed a battery of tasks that assessed EFs, and then were randomly assigned to one of the four training groups, and completed a 21-day adaptive procedure that targeted a specific sub-component of EF (or was comparatively engaging and challenging, but did not train a specific EF). At post-test, participants returned to the lab to repeat the battery of EF tasks. Using this design, we were able to compare performance before and after training to examine direct training effects (i.e., improvement on the trained task), near-transfer effects (i.e., improvement on a different task that measures the same construct), and far-transfer effects (i.e., improvement on a different task that measures a different construct) in each training group.

In line with our predictions, all four training groups showed some evidence of direct training; performance on the trained task improved from pre- to post-training. In the WM and CF training groups, this improvement was evident both in terms of their accuracy and difficulty level achieved, suggesting that repeated practice on the training task enhanced efficiency and ability in the trained EF measure. In the IC and active control groups, repeated practice on the SST/LD task helped participants to achieve higher levels of difficulty (i.e., to ignore an increasing number of competing stimuli/distinguish lower frequency words and non-words with more retained syllables), but did not improve their overall accuracy. It is possible that improvements in the IC training group were observed only on levels achieved and not accuracy because the task was too easy for this young adult sample, and therefore, the lack of significant improvement in accuracy might reflect a ceiling effect in performance (accuracy for hits averaged 98% at pre-training in this group). Future studies could adopt an ageing sample or clinical population who are likely to show more impaired performance at baseline, and therefore have more capacity for improvements through EF training. Overall, these effects are consistent with previous research, showing that practice improves performance on the trained task (Noack et al., 2009; Stine-Morrow & Basak, 2011).

In contrast, evidence for near transfer between different tasks that measured the same EF was very weak, and none of the effects reached statistical significance. These findings contrast with previous studies that have shown near-transfer improvements following WM training (Heinzel et al., 2014, 2016; K. Z. Li et al., 2010; Maraver et al., 2016; Thorell et al., 2009), IC training (Berkman et al., 2014; Enge et al., 2014; Thorell et al., 2009), or CF training (Karbach & Kray, 2009). Importantly, however, our near-transfer assessment tasks were specifically chosen to use different paradigms and stimuli to those used in the corresponding EF training task (i.e., WM was assessed with an OSpan task and trained with an *n*-back task; IC was assessed with a Stroop task and trained with an SST; CF was assessed with the WCST and trained with a colour/shape switching task). This was done to isolate transfer effects on the cognitive process itself, and to avoid carry-over effects from shared strategies or response requirements between tasks. In fact, training benefits in previous research are strongest when the demands of the transfer task are highly similar to the trained task (e.g., Brehmer et al., 2012; Harrison et al., 2013; Soveri et al., 2017), and effects are much less consistent when transfer tasks impose different processing demands (e.g., Blacker et al., 2017; Gathercole et al., 2019; Minear et al., 2016). These observations and the finding that near-transfer effects were not evident in any of the three sub-components of EF tested here support the view that training effects seen in previous research most likely reflect specific features of the trained/assessment tasks and cognitive routines learnt during training, rather than more fundamental training benefits to the underlying cognitive ability (Gathercole et al., 2019). The specificity of near-transfer effects has been highlighted by two recent studies. Holmes et al. (2019) systematically manipulated the paradigm (*n*-back or complex span) and stimuli (verbal or visuo-spatial) used in a WM training programme, and reported no transfer effects between paradigms, even when stimuli were matched. Similarly, Byrne et al. (2020) systematically tested the boundary conditions for near-transfer training benefits within and across WM paradigms with different categories of stimuli, and found paradigm-specific improvements following training that did not extend across different WM paradigms.

Similarly, evidence for far transfer between the different sub-components of EF tested here was weak, and none of the significant improvements in pre- to post-training performance in individual groups was reflected in a higher order interaction between Time and Training group or differed from the practice-based improvement in the control group, so must be considered with caution. WM training did not lead to any improvements on tasks that measured IC or CF, nor did IC training or CF training alter performance on measures of CF or IC, respectively. However, some evidence for far transfer between sub-components of EF was evident; training in IC or CF led to significant improvements on a measure of WM (OSpan), though only the IC training group improvement would withhold adjustment for multiple comparisons. The finding that WM ability might be enhanced indirectly by training other sub-components of EF mirrors results seen in previous research (e.g., Hasher et al., 2007; Karbach & Kray, 2009) and could be indicative of genuine transfer of a cognitive ability and specialisation of its underlying brain regions. However, it is important to note that success on the OSpan task used to assess WM here relies on multiple cognitive processes (i.e., it is not a “pure” measure of WM), and that the necessary response strategies share some of the same features as those practised in the IC and CF training tasks. Specifically, in addition to the broad demands on WM updating and maintenance, the OSpan task requires participants to rapidly switch between the distractor maths problem and rehearsal of the memory items (Towse et al., 1999), and to suppress irrelevant information from the maths problems (Towse et al., 2000). Therefore, in line with the specificity accounts discussed above, it is likely that these limited far-transfer effects are due to the broader EF skills that were practised within the specific IC and CF training paradigms, and reflect overlapping cognitive routines that were developed during training (Gathercole et al., 2019). This interpretation is further supported by the fact that performance on the OSpan task did not improve significantly following WM training on the *n*-back task, which shares many of the general WM processes as the OSpan task (i.e., maintenance, updating and memory search processes), but differs in terms of its sub-routine processes. The sample size used in the current experiment is large compared with most other training studies, and therefore should have been sufficient to detect relatively small effects of training if they existed. These findings are therefore consistent with previous meta-analyses in showing limited evidence for far transfer of skills between sub-components of EF (e.g., Kassai et al., 2019), and highlight the importance of cognitive training routines that include more than a single component of EF to observe gains that transfer effectively to benefit individuals in clinical or educational settings. Finally, we note that improvements in performance at the post-training session for the OSpan, IC, and CF tasks are likely to be influenced by a test–retest advantage.

Taken together, this research suggests that participants learned to perform particular cognitive tasks (i.e., direct training), but this did not lead to improvement in latent cognitive abilities underling these tasks (e.g., task switching, response inhibition), and therefore contributes to theoretical debates on cognitive training and transfer effects. Transfer effects are thought to occur when the skills learnt in one domain generalise to enhance performance in another domain, and the degree to which this transfer occurs is directly related to the extent of shared features between the trained and untrained task (Byrne et al., 2020; Singley & Anderson, 1989; Woodworth & Thorndike, 1901). This study addressed a gap in the literature by comparing training effects directly within and between different domains of EF (WM, IC, and CF) in a single study; the majority of previous EF training research has focused on understanding the boundaries of training effects in a single domain of EF, most frequently WM. Because we tested transfer effects using different paradigms for training and assessment, even within a sub-component of EF, our study was able to isolate any training benefits on the core EF skills without scaffolding from the paradigm-specific cognitive routines learnt during training. Transfer processes are mediated by neural plasticity, as quantifiable changes emerge in the cortical and sub-cortical brain areas that subserve the trained cognitive ability through practice (Dahlin et al., 2008). The shared features view predicts that near-transfer effects are more likely and stronger than far-transfer effects because in the former, the trained and untrained abilities share more common features (i.e., should rely on related cognitive and neural mechanisms). More recently, researchers have emphasised the role of the learning context, and its interaction with the content of the learned ability, suggesting that transfer effects across domains depend on the success of applying principles or strategies that are shared between the different tasks (Barnett & Ceci, 2002; Gathercole et al., 2019). As such, transfer across cognitive domains relies on participants learning new skills that can be applied in similarly structured tasks. The finding in the current experiment that indirect training benefits were absent following training in the *same* sub-component of EF (i.e., near transfer), but showed some benefits following training in a *different* sub-component of EF (i.e., far transfer) goes against traditional shared features accounts of transfer which would propose a greater advantage when there is greater overlap between cognitive abilities. As noted above, the weak evidence for far transfer from IC training to WM capacity is likely due to paradigm-specific overlap between the training and assessment tasks or that the training task activated multiple components of EF, which suggests that transfer effects are mediated by cognitive routines learned during training and shared with the transfer task rather than pure enhancement of the underlying EF process. Thus, the results presented here reinforce the proposal that cognitive training is tightly bound by the paradigm being used and that transfer within and across EF domains in the absence of paradigm overlap is limited or non-existent, despite the high degree of correlation between EF sub-components (Friedman et al., 2006; Miyake et al., 2000). Further research is needed to test how far training effects can transfer when shared cognitive routines are used to reinforce learning of the novel task.

Finally, we note some limitations with the current experiment and propose some important avenues for future research in this area. First, some of the training tasks may not have been challenging enough for our highest performing participants, meaning that a ceiling level was reached (as seen in the SST training task accuracy), and the crucial adaptive aspect of the training was not consistent across participants/groups. Relatedly, the four training tasks differed in the number of adaptive levels included (ranging from eight to 15) and the degree to which increasing difficulty between levels was matched between training tasks, which may have limited comparability between training groups. In addition, the average level measure was not based on an objective level of difficulty on the task (though it has been used in previous training studies, e.g., Chooi & Thompson, 2012; Colom et al., 2013; Jaeggi et al., 2008; Kuper & Karbach, 2016; Von Bastian et al., 2013); thus, we must be cautious in interpreting increases in level achieved as direct evidence for reduced difficulty in the training tasks.

The current tasks were selected based on those most commonly used in the field of cognitive training, and to avoid overlapping procedural elements between tasks. However, it is noted that some of the specific tasks used here are likely to have activated multiple sub-components of EF and are therefore limited in terms of cognitive specificity (as discussed for the OSpan task above). For instance, previous research has highlighted issues with using the Stroop task as a near-transfer measure of IC because it is more complex than most other measures of IC, requiring high levels of cognitive control to manage attention and semantic processing (Enge et al., 2014). Building on the current research, future studies should aim to take a more systematic approach to controlling for similarities/differences in sub-routines between tasks to isolate key components that lead to training effects (as in Holmes et al., 2019). Importantly, our experiment tested a young adult student population, who are at their peak of cognitive functioning (Diamond, 2013; Hartshorne & Germine, 2015), and therefore, the results may not be generalisable across the general population. Notably, previous research has observed larger training gains in groups whose cognitive abilities are not at their peak, e.g., among older adults (Karbach & Verhaeghen, 2014), children (Zhao et al., 2018), or clinical groups (e.g., Hallock et al., 2016; Holmes et al., 2010; Leśniak et al., 2020). Finally, although conducting a more systematic and paradigm-specific experimental procedure helps to isolate different cognitive functions that might lead to transfer, it has been suggested that to see widespread benefits of training on cognitive capacities, diverse skills must be practised. Future studies could adopt more varied training programmes that tap multiple processes within a specific sub-component of EF (e.g., maintaining, updating, and recalling in WM), while reducing the contributions of other EF sub-components, to allow a more rigorous exploration of “brain training” and its generalisability across wider domains of functioning (e.g., social interaction; Kloo & Perner, 2003; Santiesteban et al., 2012).

In sum, we conducted a pre-registered experiment that sought to adopt a robust approach to EF training programmes recommended by Simons et al. (2016), and investigated the efficacy and generalisability of EF training within and between three sub-components of EF (WM, IC, and CF) compared with an active control training programme. In line with previous literature, we found robust direct training effects, but limited evidence to support near- and far-transfer effects (Heinzel et al., 2014, 2016; Owen et al., 2010). Where indirect training benefits emerged, they were statistically weak and the effects were more readily attributable to overlapping training/assessment task routines and test/re-test effects, rather than more general enhancements to the underlying cognitive processes or neural circuits. Further research is needed to isolate sub-components of EF targeted in training programmes, while systematically manipulating paradigm-specific commonalities between tasks. Such an approach would allow researchers to further explore what kinds of training most reliably lead to performance changes, and to assess the generalisability and specificity of training effects on cognition and beyond.

## Supplemental Material

sj-docx-1-qjp-10.1177_17470218211002509 – Supplemental material for Training executive functions using an adaptive procedure over 21 days (10 training sessions) and an active control group Click here for additional data file. Supplemental material, sj-docx-1-qjp-10.1177_17470218211002509 for Training executive functions using an adaptive procedure over 21 days (10 training sessions) and an active control group by Martina De Lillo, Victoria EA Brunsdon, Elisabeth EF Bradford, Frank Gasking and Heather J Ferguson in Quarterly Journal of Experimental Psychology

## Notes

1. We allowed a minimum of 20 days and a maximum of 28 days between pre- and post-training sessions (*M* = 21.4, *SD* = 1.1).
