# Supplementary material for: Training executive functions using an adaptive procedure over 21 days (10 training sessions) and an active control group
Source: Q J Exp Psychol (Hove). 2021 Mar 30;74(9):1579–94. doi: 10.1177/17470218211002509 (PMC8358555; doi:10.1177/17470218211002509)
Supplement: sj-docx-1-qjp-10.1177_17470218211002509 – Supplemental material for Training executive functions using an adaptive procedure over 21 days (10 training sessions) and an active control group [file sj-docx-1-qjp-10.1177_17470218211002509.docx]

**SUPPLEMENTARY MATERIAL for**

**Training executive functions using an adaptive procedure over 21-days (ten training sessions) and an active control group**

Martina De Lillo

Victoria E.A. Brunsdon

Elisabeth E.F. Bradford

Frank Gasking

Heather J. Ferguson

School of Psychology, University of Kent, U.K.

Figure A: Average z-scored accuracy achieved during the pre- and post-training sessions and the ten online training sessions, plotted for each training group. Note that accuracy for each task is calculated as proportion of Hits minus False Alarms.

**Inhibitory Control (SST-Flanker) Working Memory (n-back)**


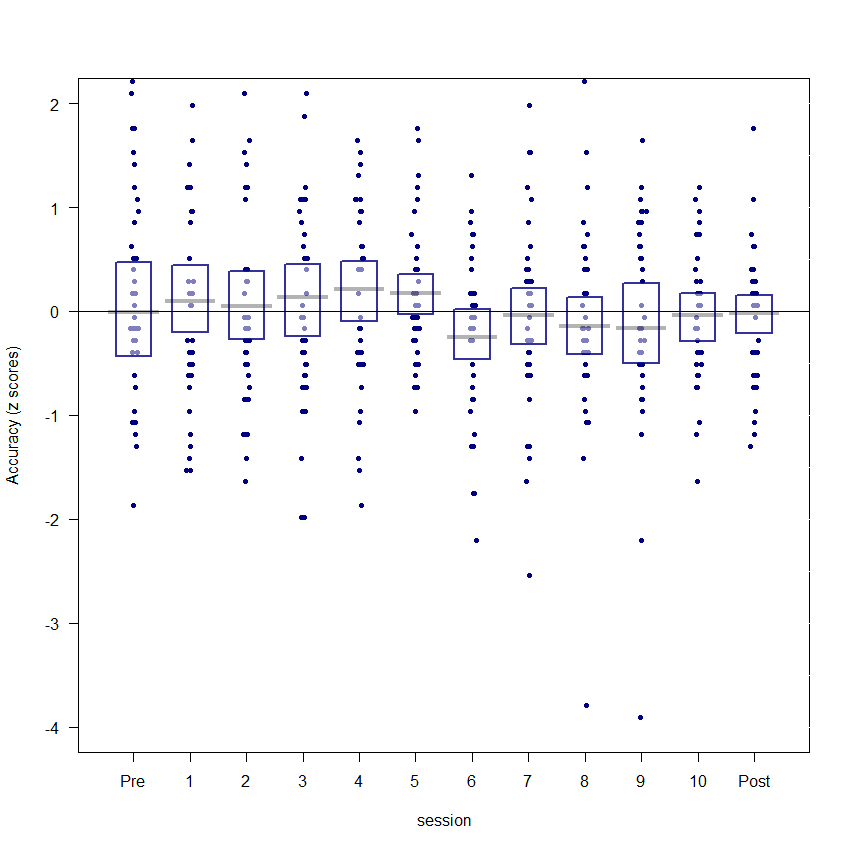

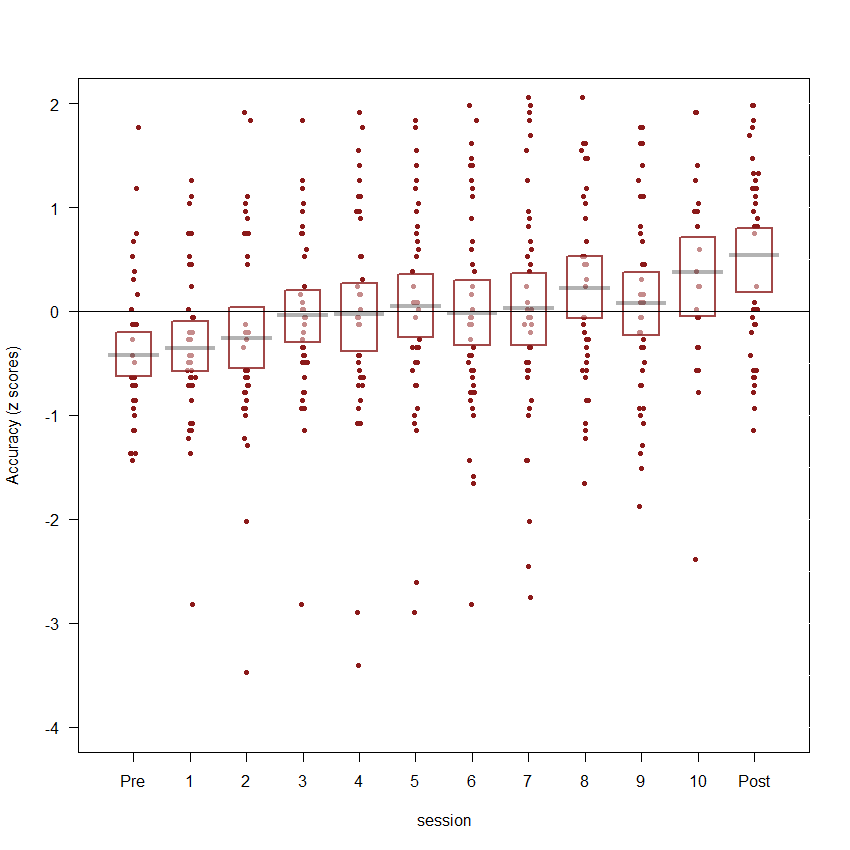


**Cognitive Flexibility (Task switching) Control (Lexical decision)**


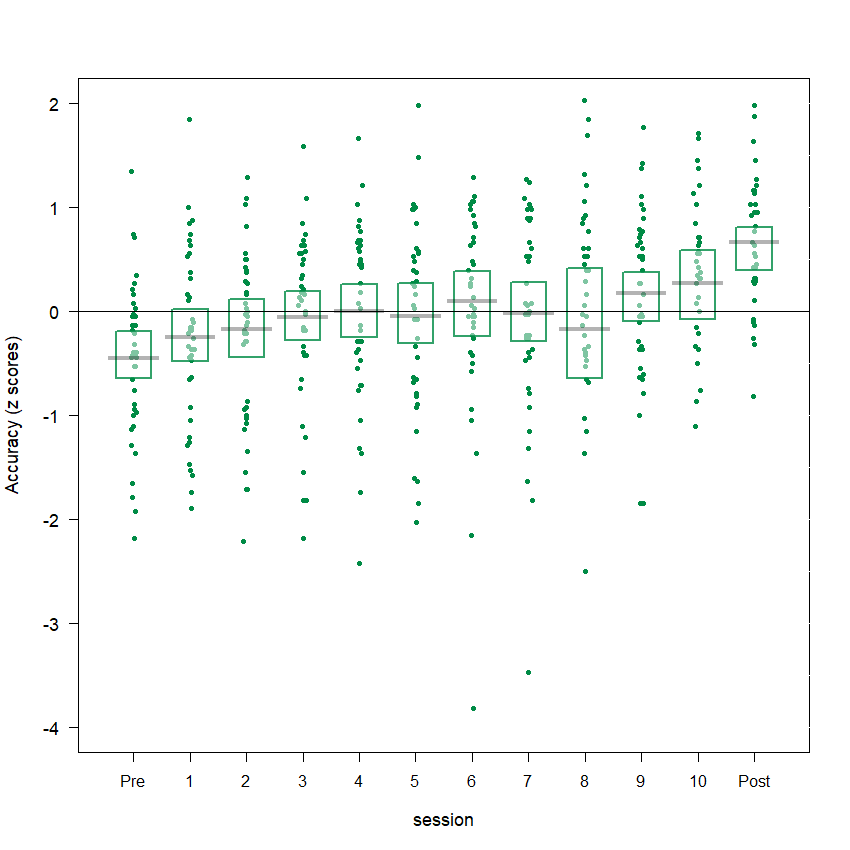

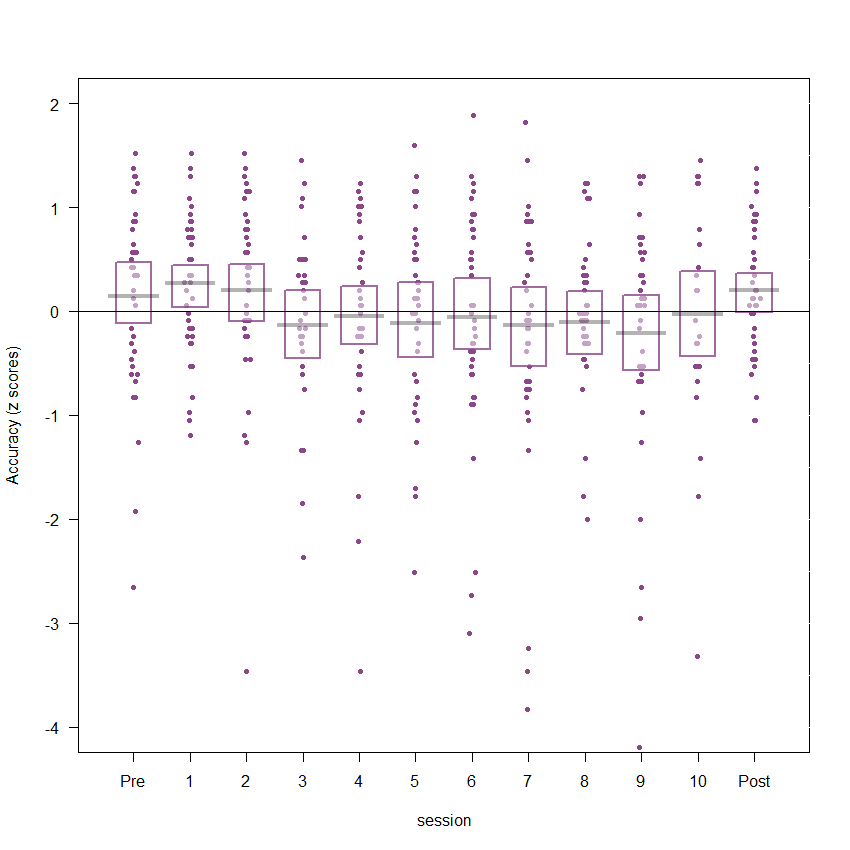


Figure B: Average z-scored level achieved during the pre- and post-training sessions and the ten online training sessions, plotted for each training group.

**Inhibitory Control (SST-Flanker) Working Memory (n-back)**


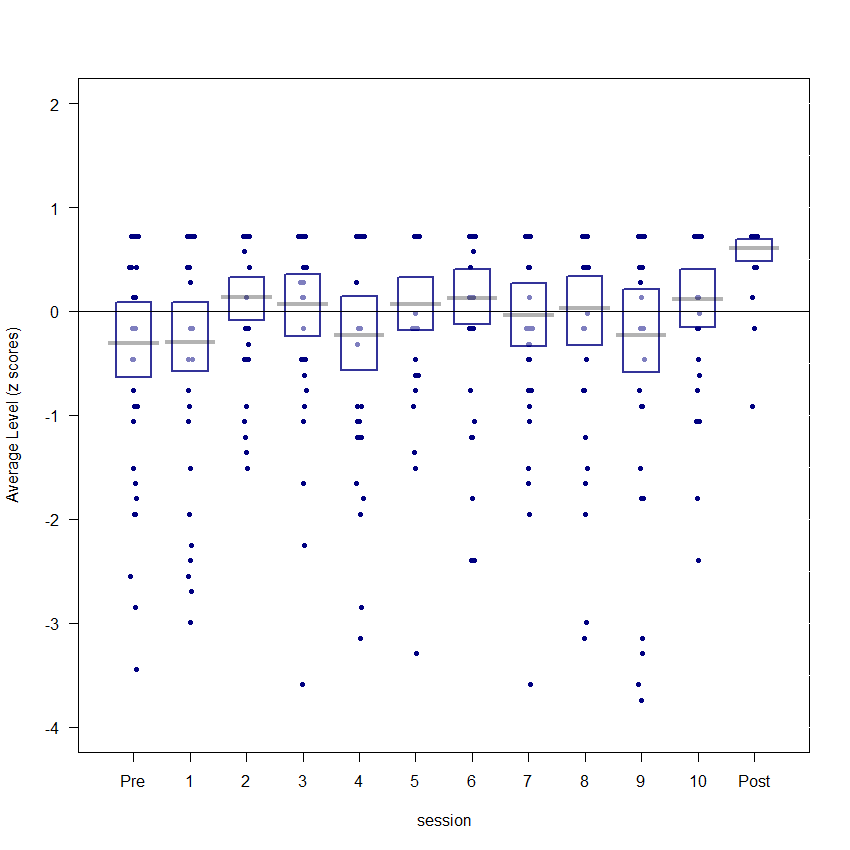

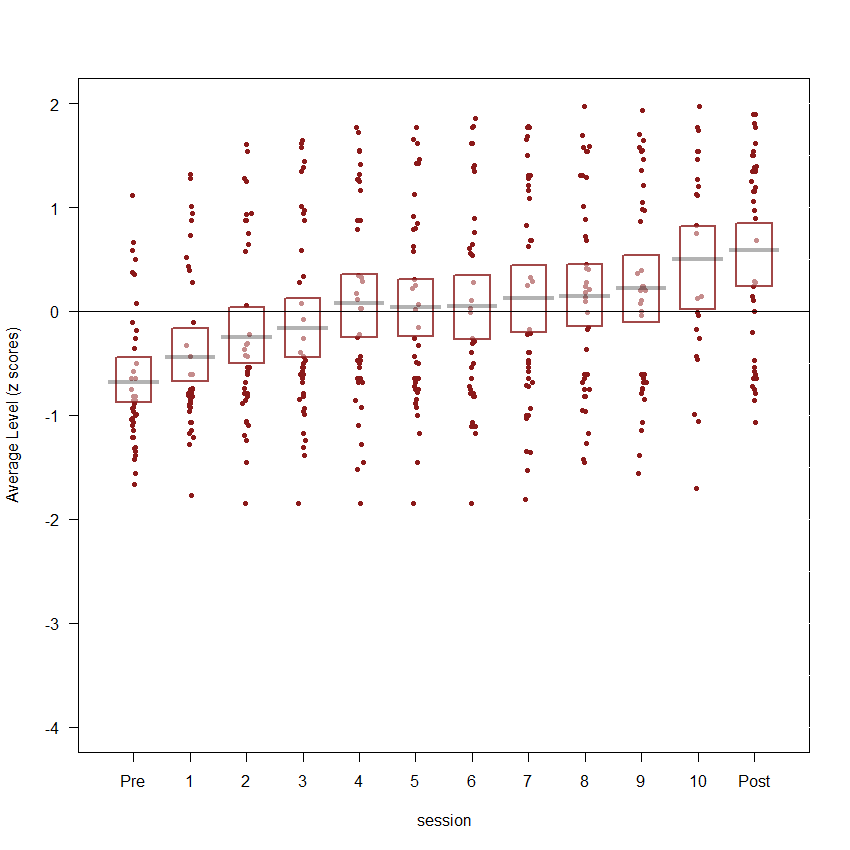


**Cognitive Flexibility (Task switching) Control (Lexical decision)**


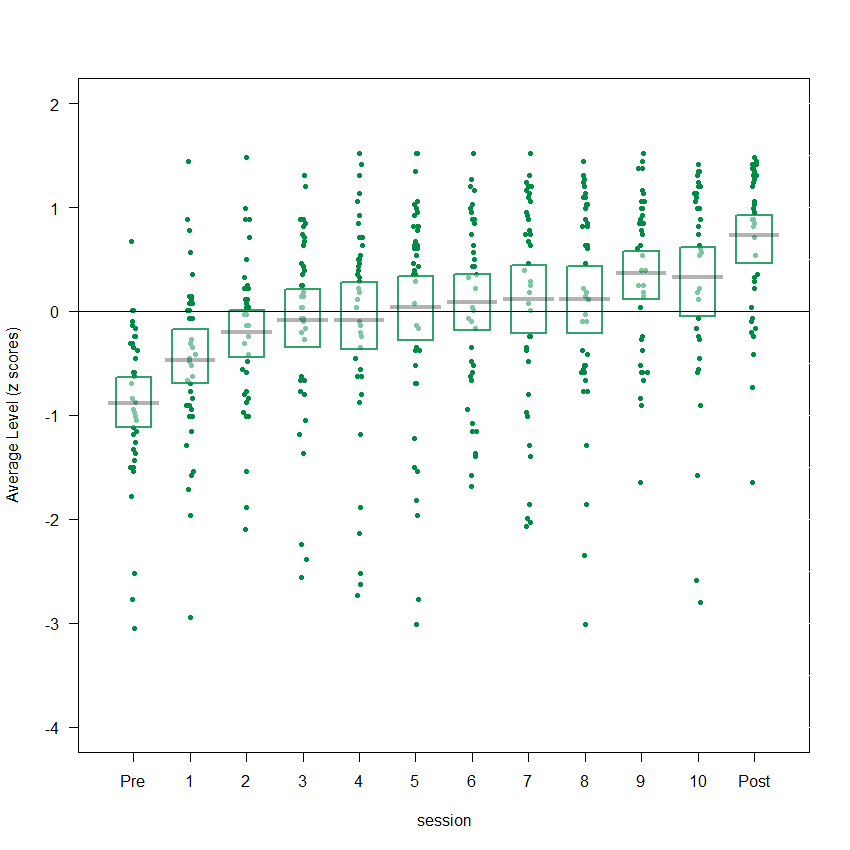

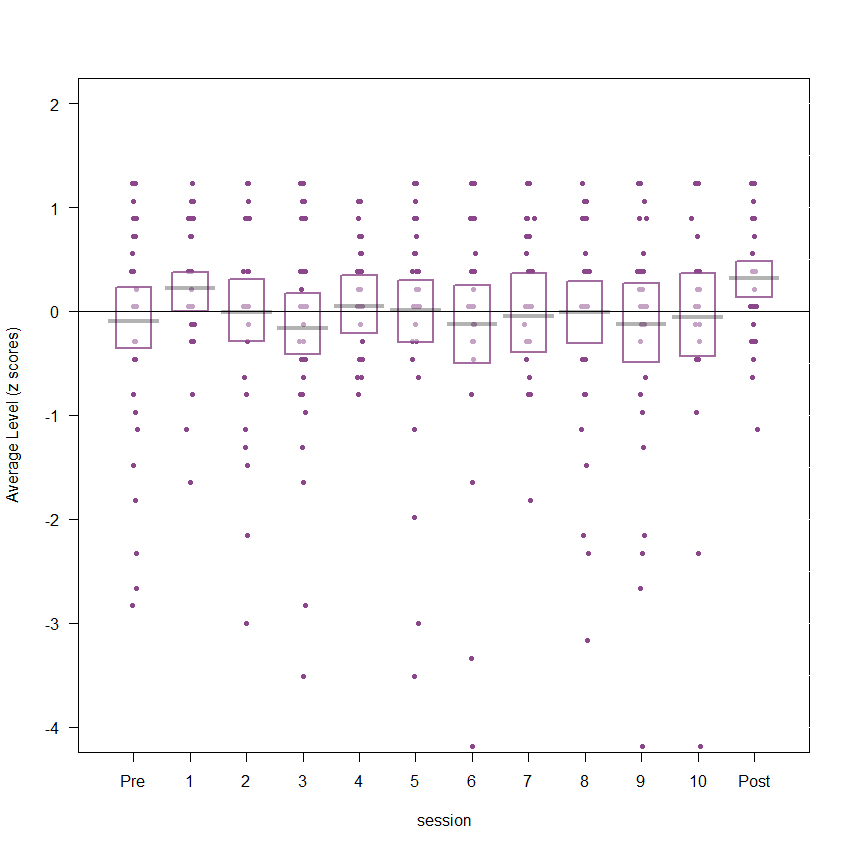


Figure C: Average raw scores for accuracy achieved during the pre- and post-training sessions and the ten online training sessions, plotted for each training group. Accuracy for each task is calculated as proportion of Hits minus False Alarms.


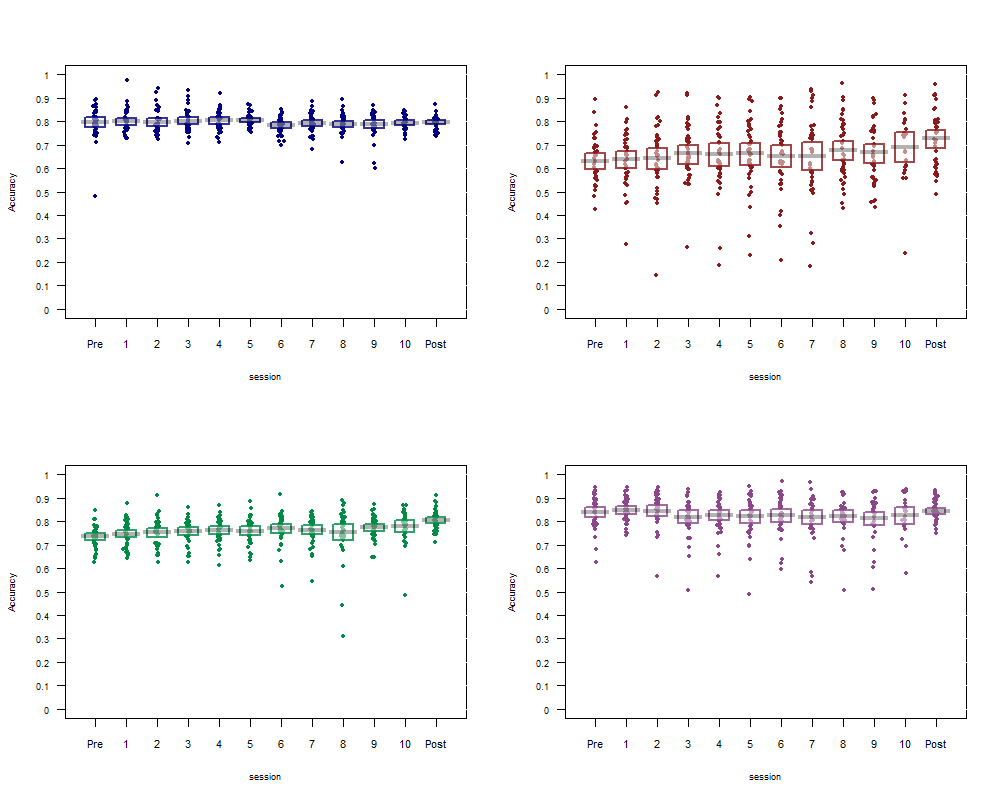


**Inhibitory Control (SST-Flanker) Working Memory (*n*-back)**

**Cognitive Flexibility (Task switching) Control (Lexical decision)**

Figure D: Average raw scores for level achieved during the pre- and post-training sessions and the ten online training sessions, plotted for each training group. Note that the number of levels differs for each task (SST = 8 Levels; NB = 15 Levels; CF = 12 Levels; LD = 8 Levels).


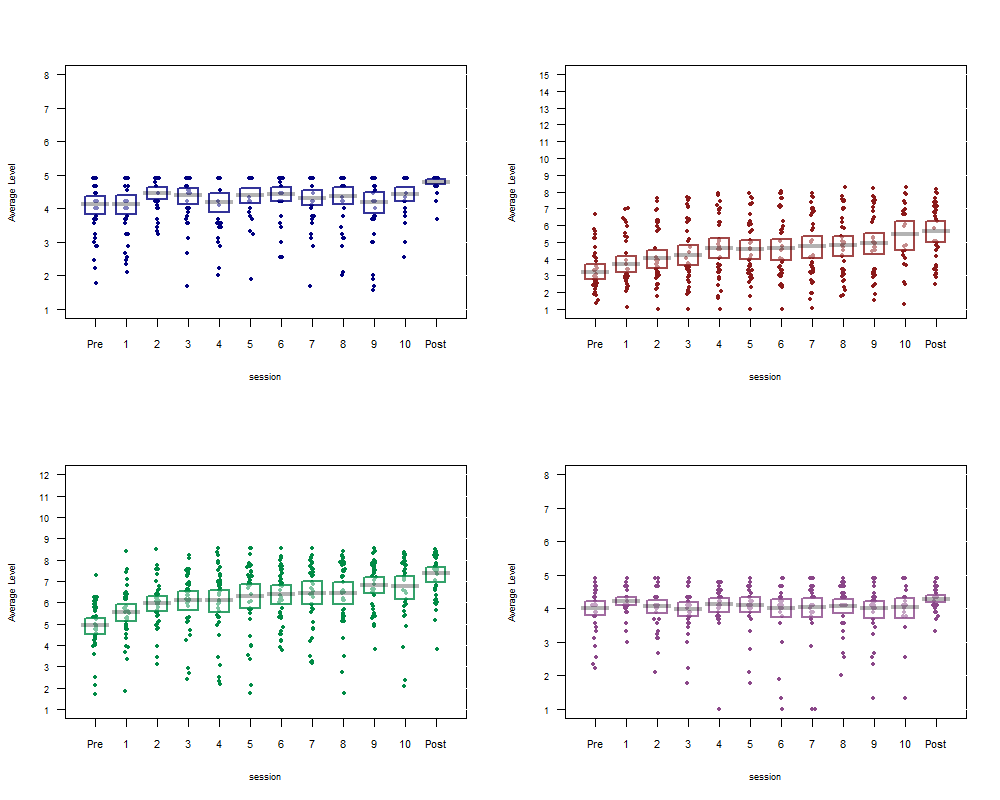


**Inhibitory Control (SST-Flanker) Working Memory (*n*-back)**

**Cognitive Flexibility (Task switching) Control (Lexical decision)**
